# Comparison of modified extended right lobe graft versus modified right lobe graft in adult living donor liver transplantation: Experience from Pakistan

**DOI:** 10.12669/pjms.40.8.7825

**Published:** 2024-09

**Authors:** Abdul Ghaffar, Kaleem Ullah, Syed Hasnain Abbas, Hafiz Bilal

**Affiliations:** 1 Abdul Ghaffar, FCPS Pir Abdul Qadir Shah Jeelani Institute of Medical Sciences, Gambat, Sindh, Pakistan; 2 Kaleem Ullah, FCPS, FACS Pir Abdul Qadir Shah Jeelani Institute of Medical Sciences, Gambat, Sindh, Pakistan; 3 Syed Hasnain Abbas, FCPS Pir Abdul Qadir Shah Jeelani Institute of Medical Sciences, Gambat, Sindh, Pakistan; 4 Hafiz Bilal, FCPS Pir Abdul Qadir Shah Jeelani Institute of Medical Sciences, Gambat, Sindh, Pakistan

**Keywords:** Liver transplantation, living donor, Modified extended right-lobe graft

## Abstract

**Objectives:**

To compare the outcomes of modified extended right lobe graft (MERLG) and modified right lobe graft (MRLG) in living-donor liver transplantation (LDLT).

**Methods:**

This retrospective study was performed at the Liver transplant department of the Pir Abdul Qadir Shah Jeelani Institute of Medical Sciences Hospital, Gambat, Pakistan, from March 2019 to September 2020. The outcomes of 20 MERLG donors and recipients were compared to those of 74 MRLG donors and recipients. Demographics, operative parameters, complications, hospital stay, and one-year survival were compared between the two groups.

**Results:**

The mean graft volume of the MERLG group was more (637.10 ± 71.35 g) than in the MRLG group (562.27 ± 57.77 g), (p= 0.001). Donor blood loss was higher in the MERLG group (680.10±170.60 ml) compared to the MRLG group (650.23±190.65 ml), p=0.527. In addition, the operative time was longer in the MERLG group (345.80±76.90 min) than in the MRLG group (318.12±100.80 min) (p= 0.257). The MERLG recipients were sicker (mean MELD score of 22.54±3.67) than the MRLG (18.86±4.37) (p=0.001). The drain output was higher in the MRLG group (1340 ± 470.32 ml) than in the MERLG group (1110 ± 450.60 ml) (P =0.045). No significant difference was found when comparing postoperative laboratory results and complications between the donor and recipient groups (p >0.05). Kaplan-Meier analysis showed a 95% one-year survival in MERLG group compared to 90.7% in the MRLG group (p=0.549).

**Conclusion:**

With appropriate technical expertise, MERLGs are technically safe and feasible in LDLT donors without any added risks. MERLGs also yielded better outcomes in sick recipients.

## INTRODUCTION

The living donor liver transplantation (LDLT) techniques have significantly improved with time. The outcome of liver transplantation is not only dependent on graft quality and volume but also on appropriate inflow and outflow techniques. Inadequate outflow leads to congestion, hepatocyte necrosis, and graft failure.^1^,^2^ Right-lobe grafts (RLGs) are considered ideal adult LDLT grafts. The venous outflow of the anterior segment of the RLGs depends mainly on the middle hepatic vein (MHV) tributaries, that is, segment V/VIII veins.^2^ Based on anterior segment drainage, the RLGs are classified into three; Partial right lobe grafts (PRLG) having only right hepatic vein (RHV), Modified right lobe graft (MRLG) having reconstructed segment V/VIII veins, creating a “neo-MHV.” In the Modified Extended Right Lobe graft (MERLG) the full-length MHV is included.^3^

Lee et al.^4^ reported anterior segment congestion (ASC), early graft dysfunction, and sepsis-related mortality in a few of the RLG recipients. So, MHV inclusion in RLGs was introduced by Lo et al. to avoid ASC. They labeled RLGs with MHV as the best-quality graft.^5^ However, MHV inclusion in RLGs has certain limitations. MHV inclusion poses certain donor risks, like segment IV congestion, more transaction bleeding, and fear of small future remnant liver (FLR). In addition, it may not always be possible to take RLG with MHV due to unfavorable factors. The decision of MHV inclusion in RLGs depends on multi-factors, which include MHV anatomy, FLR, GRWR and surgeon expertise.^1^ To avoid donor risks and ASC in RLGs, various centers started reconstruction of Segment V/VIII veins creating neo-MHV.^1^

However, at times the reconstruction of these major MHV tributaries may not be sufficient to avoid ASC. Also, the reconstruction of MHV tributaries with diameters <3 mm remains challenging.^2^,^5^ Moreover, a study reported sixty-two percent ASC in RLGs even with segment V/VIII veins reconstruction.^6^

Few authors claim that despite ASC, the graft regenerates sufficiently in the posterior segment, which fulfills the patient’s metabolic demands. However, few patients may behave differently and can develop small-for-size syndrome and graft dysfunction. Few others reported that MERLG is vital for high MELD recipients.^7^,^8^ Also, the MERLG provides a relatively bigger sized graft compared to MRLG which may be beneficial for recipients with low calculated GRWR.^9^,^10^

Despite multiple studies, MHV management in adult LDLT remains controversial. In addition, whether the efficacy of neo-MHV is as good as that of native MHV remains unclear. Moreover, the impact of MHV inclusion in RLGs, on donor morbidity requires further investigation. In this study, we retrospectively reviewed our experience of harvesting RLGs with MHV in adult LDLT recipients. We also aimed to determine donor safety and recipient outcomes by comparing them to the MRLG group. This is the first national study to report RLGs and MHV outcomes.

## METHODS

This retrospective study was conducted on selected LDLT donors and recipients, whose procedures were performed between July 2019 and September 2020 at the liver transplant department of Pir Abdul Qadir Shah Jeelani Institute of Medical Sciences, Gambat, Pakistan. Over 15 months, 157 ABO-compatible LDLT procedures were performed. Of these 94 pairs, either the MRLG or MERLG were included in this study (74 with MRLG and 20 with MERLG). The PRLG and left lobe grafts, LDLT procedures which were performed for acute liver failure (ALF), acute on chronic liver failure (ACLF), those who received a portal jump graft, and re-transplanted cases were excluded due to their complicated disease/procedure for study purposes. The selected donor and recipient pairs were divided into two groups; the MERLG and MRLG groups. Twenty LDLT pairs were found in the MERLG group and 74 in the MRLG group. Both groups were compared in terms of demographics, operative parameters, graft characteristics, postoperative labs and complications, length of hospital stay, and one-year survival.

### Ethical Approval:

Approval for this study was obtained from the hospital ethical committee (Reference No.: IRB/22/15 Dated October 12, 2021).

### Pre-Transplant work up:

The recipients’ pre-transplant workup and detailed donor selection protocol/criteria have been described elsewhere.^11^ Informed video consent for the LDLT procedure and research purposes was obtained from all the donors and recipients. The calculated GRWR for the recipients was maintained at > 0.8.

### Middle Hepatic Vein Inclusion Protocol:

The anatomy of MHV tributaries and the dominance of MHV/RHV in the RLGs were studied carefully on a Triphasic CT scan for recipients with a low calculated GRWR and high MELD score (>20). Graft procurement with MHV was considered if the donors had favorable venous anatomy, that is independent segment IV-a drainage into the LHV and FLR of >35.

### The technique of Modified and Modified Extended Right lobe grafts:

**Fig.1:**
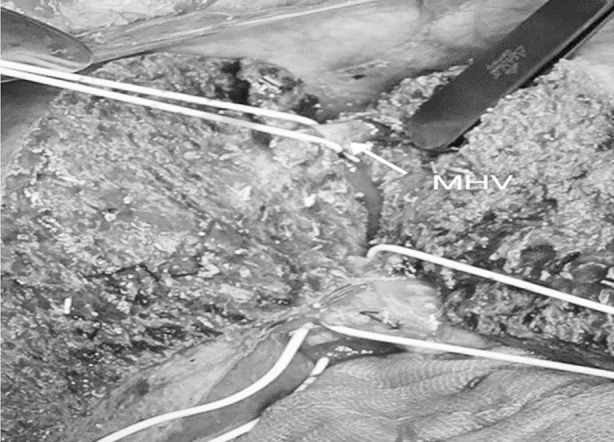
Donor hepatectomy exposing Middle Hepatic Vein (MHV).

**Fig.2:**
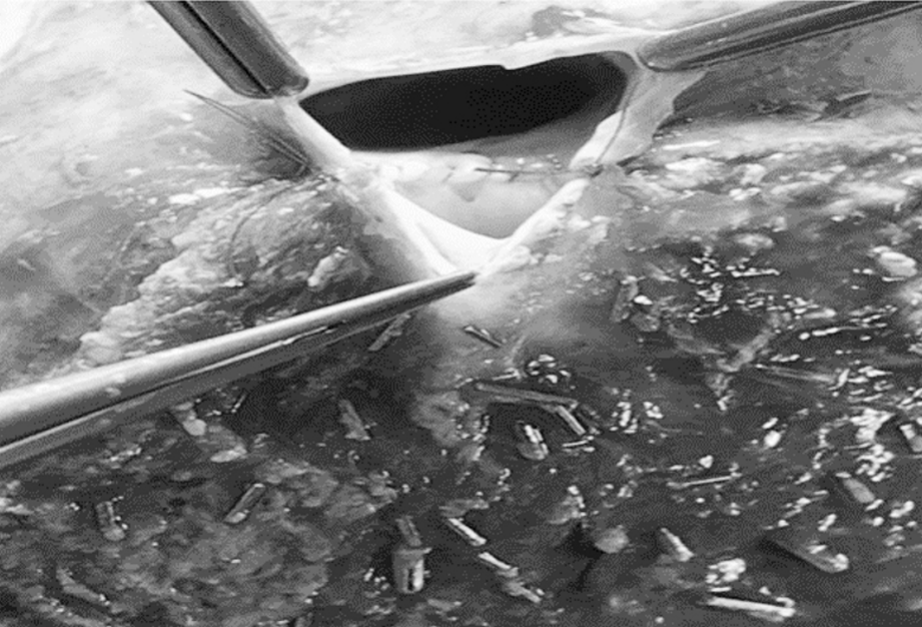
RHV and MHV Venoplasty.

During donor surgery, in the MRLGs, the transaction line was kept on the right side of the MHV, and the segment V/VIII veins were individually treated. Segment VIII/V veins (diameter >5 mm) were reconstructed on the back table with a synthetic PTFE vascular conduit with 6/0 Prolene suture in a continuous fashion, creating a neo-MHV. The neo-MHV was then anastomosed with the RHV to obtain a common orifice. In contrast, the MERLG transaction line was kept on the left side of the MHV. The MHV was divided at its entrance into the IVC (Fig.1). MHV and RHV venoplasty were done on the back table to obtain a single orifice (Fig.2).

### Recipient Surgical Technique:

Implantation was performed in the recipient using partial IVC clamping. Duct-to-duct biliary anastomosis was performed in an interrupted manner. Vascular patency including portal and arterial inflow and hepatic venous outflow were confirmed on the intraoperative Doppler ultrasonography.

### Postoperative Management and Follow-up:

Postoperatively, complete blood count and liver and renal function tests were performed daily for all donors. Donors were routinely discharged on the 5^th^ postoperative day (POD) and were followed up weekly for one month, monthly for six months, and three monthly for one year. At each follow-up, physical examination and labs, including CBC, LFTs, and ultrasound liver, were performed.

Similarly, recipients were managed in the ICU with a policy of next-day extubation. Immunosuppressant Induction was done with intravenous Methylprednisolone 500 mg intraoperatively while for maintenance tacrolimus and oral prednisolone were used. The prednisolone dose was tapered over three months. Complete blood count, liver and renal function tests, and hepatic vascular Doppler studies were performed daily for the first five PODs and then per need. Recipients were discharged routinely on the 10^th^ POD and were then followed up weekly for one month, fortnightly for three months, and monthly until the end of the 1^st^ year. On each follow-up routine laboratory tests, and tacrolimus levels were obtained.

### Data Collection:

Data were collected from the donor’s and patient’s daily charts and were maintained on the prospectively held SPSS database.

### Statistical Analysis:

The data were retrospectively analyzed using SPSS version 21. For quantitative variables, the arithmetic mean was calculated and percentages were calculated for qualitative variables. Qualitative variables such as hepatic arterial and portal vein thrombosis, and biliary complications were compared between the two groups using Chi-Square or Fischer Exact Tests. The independent sample t-test was used to compare quantitative variables, such as mean hospital stay, total bilirubin, albumin, ALT, ALT & INR values, and drain output. The one-year survival of patients in the two groups was calculated and analyzed using the Kaplan–Meier survival graph. Statistical significance was set at p < 0.05.

**Graph.1:**
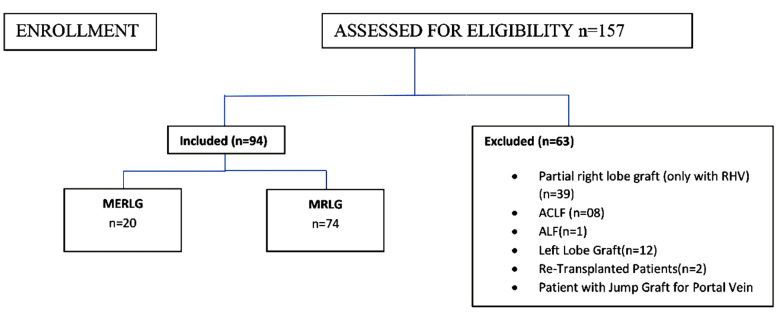
CONNSORT 2010 Flow Diagram.

## RESULTS

A total of 157 LDLT pairs were assessed for eligibility out of which 94 fulfilled the inclusion criteria, 20 in MERLG, and 74 in the MRLG group (Fig.1).

### Donors’ demographics and Graft Characteristics:

The mean age of donors in MERLG was 19.80 ± 2.11 years and 19.45 ± 4.37 years in MRLG (p= 0.73). Details of donor demographics are provided in Table I. The mean LAI of donors in MERLG was 11.26 ± 3.90 as compared to 9.87 ± 2.76 in MRLG (p=0.072). The mean FLR of donors in MERLG was 36.87 ± 2.11 as compared to 33.45±3.78 in MRLG (p = 0.001). The mean graft volume in the MERLG group was 637.10 ± 71.35 g and 562.27 ± 57.77 g in the MRLG group (p= 0.0001). Mean operative donor blood loss was 680.10±170.60 ml in the MERLG group and 650.23±190.65 ml in the MRLG group (p= 0.527). No significant difference was found when comparing parameters, such as mean warm and cold ischemia times and mean donor operative time (Table-I).

**Table-I:** Comparison of mean values of demographic and operative parameters of donors and recipients.

Variable	MERLG (N=20)	MRLG(N=74)	*P*-value
***Donors***			
Age(years)	19.80±2.11	19.45 ±4.37	0.73
Gender (Male/Female)	10/10	42/32	0.590
BMI kg/m2	21.40±2.99	22.54±3.06	0.141
LAI	11.26 ± 3.90	9.87 ± 2.76	0.072
FLR %	36.87±2.11	33.45±3.78	0.001
GRWR	0.90±0.45	1.11±0.25	0.007
Actual graft weight, gram	637.10 ± 71.35 g	562.27 ± 57.77	0.001
Warm ischemic time, minutes	32.54±10.13	36.47±8.53	0.083
Cold Ischemic time, minutes	10.59±5.02	11.63±4.28	0.355
Blood loss, ml	680.10±170.60	650.23±190.65	0.527
Operation time, minutes	345.80±76.90	318.12±100.80	0.257
***Recipients***			
Mean Age, years	43.85±10.91	39.21±10.92	0.095
Male/Female	19/1	55/19	0.023
BMI, kg/m2	23.99±3.87	24.34±5.91	0.803
HCC	3 (15%)	7(9.45%)	0.475
MELD Score	22.54±3.67	18.86±4.37	**0.001**
Blood loss (ml)	1550.78 ±550.90	1300.32±650.43	0.119
Operative time(minutes)	430.91±100.20	470.65±110.70	0.150

***Abbreviations:*** (BMI, Body Mass Index [Kg/m^2^]; LAI, Liver attenuation index; FLR, future liver remnant; HCC, Hepatocellular carcinoma; MELD, Model of End-Stage Liver Disease; GRWR, Graft-to-Recipient Weight Ratio).

### Recipient’s demographics:

In the MERLG group, 19 (95%) were male while in MRLG, 55(74.32%) were male. The mean age of recipients with MERLG was 43.85±10.91 years and 39.21±10.92 years in the MRLG group (p=0. 095). The primary etiology of end-stage liver disease was chronic viral hepatitis in 16 (80%) and 60 (81.08%) patients in the MERLG and MRLG groups, respectively. The mean GRWR in MERLG was 0.90 ± 0.45 and 1.11 ± 0.25 in MRLG (p= 0.007). No significant difference was found when comparing various recipient variables such as mean blood loss and operative time in both groups. The mean MELD score was 22.54±3.67 in patients who received MERLG while 18.86±4.37 and MRLG (p=0.001), respectively (Table-I).

The donor’s 5^th^ POD laboratory parameters were comparable. The mean 5^th^ POD drain output was 101.67 ± 56.10 ml in MERLG and 98.01 ± 34.81 ml in the MRLG group (p=0.065). The mean hospital stay of donors in MERLG was 6.35±2.40 and 5.94±2.17 days in MRLG, (p>0.05), (Table-II). A comparison of the 7^th^ POD recipient’s laboratory parameters in both groups did not show any significant differences. The MERLG recipients’ mean 7th POD drain output was lower (1100.53±450.60 ml) than the MRLG group (1340±470.32 ml), p= 0.045. The mean hospital stay was also comparable between the groups (p= 0.332), (Table-II).

**Table-II:** Comparison of Mean postoperative Biochemical parameters of Donors and Recipients.

Variables	MERLG	MRLG	P-value
***Donors***			
5^th^POD AST(IU/L)	88.84±32.63	73.24±26.56	0.029
5^th^POD ALT(IU/L)	99.01±39.29	90.93±24.09	0.254
5^th^ POD INR	1.40±0.23	1.20±0.41	0.040
5^th^POD Bilirubin, (mg/dL)	1.98±0.90	1.45±0.31	0.001
5^th^POD Drain output(mL)	101.67±56.10	98.01±34.81	0.718
Hospital stay (days).	6.35±2.40	5.94±2.17	0.465
***Recipients***			
7^th^POD AST(IU/L)	70.05±52.10	93.11±65.35	0.149
7^th^POD ALT (IU/L)	97.38±72.90	137.27±99.23	0.097
7^th^POD Bilirubin(mg/dl)	2.17±1.31	2.90±1.88	0.107
7^th^ POD INR	1.98±1.5	1.87±1.39	0.758
7^th^ POD Drain output(ml)	1110±450.60	1340±470.32	0.045
Hospital stay(days)	12.25±4.87	13.19±3.50	0.332

***Abbreviations:*** (ALT, Alanine Aminotransferase; AST, Aspartate Aminotransferase; INR, International Normalized Ratio;)

Three (15 %) and 12 (16.21 %) donors in the MERLG and MRLG groups, respectively, developed complications. These complications were sub-grouped using the Clavien-Dindo classification. Most complications were minor (Table-III).

**Table-III:** Comparison of postoperative donor complications (Clavien Dindo Grading).

Grade	Complications	MERLG	MRLG	P value
01	Surgical site infections	1(5%)	4(5.4%)	0.98
02	Wound infections needed antibiotics	0	2(2.7%)	0.45
	High drain output	1(5%)	2(2.7%)	0.604
3a	Pleural effusion required thoracocentesis	1(5%)	3(4 %)	0.852
	The abdominal collection needed percutaneous drainage	0	2(2.7%)	0.457
3b	Re-laparotomy for bleeding	0	1(1.35%)	0.601

**Fig.3:**
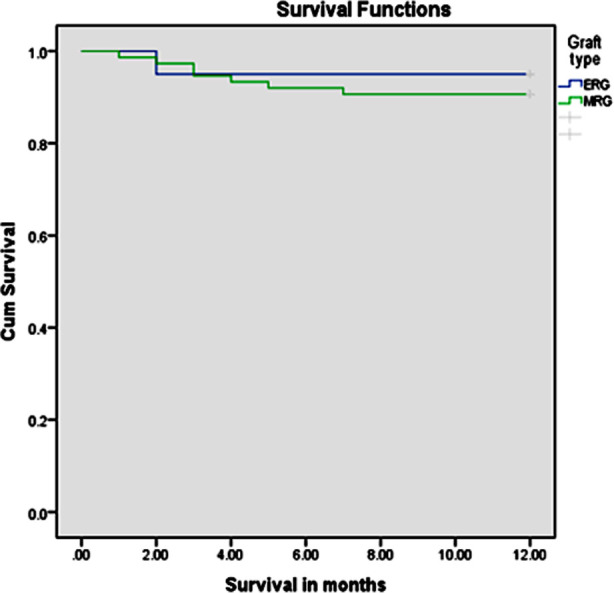
Kaplan-Meier Survival Analysis showing one-year survival between grafts with MERLG vs MELG.

No statistically significant difference was found while comparing various post-operative complications in recipients like hepatic artery thrombosis (HAT), portal vein thrombosis (PVT), biliary leakage, acute cellular rejection, and sepsis in both groups (p-value >0.05). The recipient’s 90-days mortality was also comparable between both groups (p value=0.638), (Table-IV). Kaplan-Meier analysis showed 95% 01-year survival in the MERLG group and 90.7% in the MRLG group (p=0.549), (Fig.3).

**Table-IV:** Comparison of postoperative complications in recipients.

Complications	MERLG (n=20)	MRLG (n=74)	P-Value
HAT	1(5%)	3(4.05%)	0.852
PVT	0	2(2.70%)	0.457
Biliary leakage	1(5%)	2(2.70%)	0.604
Biliary Stricture	3(15%)	10(13.51%)	0.864
Acute Cellular Rejection	2(10%)	6(8.1%)	0.787
Sepsis	2(10%)	7(9.5%)	0.941
90 days Mortality	1(5%)	6(8.1%)	0.638

***Abbreviations:*** (HAT), Hepatic artery thrombosis; PVT, portal vein thrombosis.

## DISCUSSION

With the standard adult LDLT grafts (RLG without MHV) the risk of ASC always remains.^4^,^10^ To avoid ASC, several technical modifications were made. Hong Kong group introduced MHV inclusion in RLGs, aiming to provide grafts having adequate drainage to fulfill the recipient’s metabolic demands.^5^ The Toronto group reported the technique of neo-MHV using interposition grafts.^12^ The segment V/VIII veins reconstruction using synthetic grafts or cryopreserved veins was then reported by others too.^4^,^13^ But, despite the neo-MHV reconstruction, the ASC was reported in a few recipients.^6^ Also, the majority of centers avoid RLGs with MHV to avoid segment IV congestion of the remnant donor liver.^10^

In the MERLG group, we harvested the graft with MHV, and in the MRLG group, a neo-MHV was reconstructed. We compared the outcome of these grafts (in donors as well as recipients). The graft volume was higher in MERLG as expected, compared to MRLG (p=0.001). We consider those grafts for MHV consideration having FLR >35%. The comparison of FLR in both groups showed greater pre-op FLR in MERLG (36.87±2.11%) than MRLG group (33.45±3.78%) (p=0.001). Although donor blood loss and operative duration were a bit higher in the MERLG group compared to the other group but were not statistically significant. The study of Tan et al. also reported a longer operative time for MHV grafts.^9^

All our donors in the MERLG group recovered smoothly and were discharged like routine donors. The possible reasons for comparable outcomes among both donor groups may be parameters like FLR (>35%), LAI >5, and younger donor age. Our center’s experience supports the other international reports that MHV inclusion in RLGs is safe.

The recipients in MERLG were found to be sicker (mean MELD Score of 22.54±3.67) compared to the MRLG (18.86±4.37), p=0.001. We harvested the graft with MHV in sick patients for better recipient outcomes. This has been reported by other studies as well.^7^,^8^

Hong Kong group shared their experience of RLGs with MHV. They reported slightly increased donor morbidity. Increased morbidity in the donor subgroup was due to a decreased regeneration capacity of the aged liver (age >40 years). They also observed an increased frequency of renal complications and graft dysfunction in those patients who received RLGs without MHV compared to patients with RLGs with MHV.^10^ Contrast to them we avoid the donors with age > 40 years. That might be the reason for less donor morbidity in MERLG in our study.

Another prospective study reported the outcome of 100 living donors’ hepatectomies, 49 RLGs with MHV and 51 without MHV. They concluded that operative parenchymal transection time was significantly longer in the MHV group.^14^ We also experience slightly longer transaction time in MHV grafts. Our calculated intraoperative blood loss was also comparable for both groups, similar to their observation. Their data also concluded that donors of MHV grafts did not experience any peak massive increase in the levels of transaminase, total bilirubin, and INR levels in the first post-op week. We also observed no significant rise in post-op LFTs in the MERLG group donors.

In certain cases, RLG with MHV overcomes the low GRWR. In certain LDLT pairs with low calculated GRWR, RLG with MHV should be considered if the donor is suitable. In this study, the mean GRWR in the MERLG group was lower (0.90±0.45) than the MRLG group recipients (1.11±0.25). For low GRWR (<0.8%) recipients, graft with MHV (MERLG) is a practical solution. This had been reported by other authors as well.^1^,^5^

Although in this study, patients in the MERLG group were more ill compared to the other group, however, their various post-operative biochemical parameters including liver enzymes, bilirubin, and INR on 7^th^ POD were comparable with the other group. Also, drain out was found lower in MERLG compared to MRLG (p=0.045). Moreover, the incidence of various postoperative complications (like biliary, vascular, and immunological) and 90 days’ mortality were comparable in both groups. One survival was also found better in MERLG. Other reports have also demonstrated that the MERLG yields a good outcome in recipients without any extra donor risk.^1^,^9^

Few investigators evaluated the patency of neo-MHV. They found a better patency rate for biological grafts compared to synthetic grafts.^15^ Although we did not study the patency of neo-MHV.

Nevertheless, this study concluded that in recipients with low GRWR or high MELD scores, MERLG is a better option for optimal outcomes. Also, it does not pose any extra donor morbidity. The decision of MHV inclusion in RLG should be based on graft anatomy paired with the recipient’s disease severity and surgical expertise.

### Limitations:

It includes the retrospective nature of the study, single-center study, and a relatively small size sample. Also, we did not study the long-term patency of neo-MHV. Further studies are suggested on this.

## CONCLUSION

The extent of donor right hepatectomy should be tailored according to the particular situations i.e., considering the metabolic demands of the recipient (recipient illness), GRWR, MHV anatomy, and FLR. In recipients with low GRWR and high MELD scores, MERLG is a better option if FLR and graft anatomy permits.

### Authors’ Contribution:

**AG:** Designed the study and did the final editing of the manuscript.

**KU:** Prepared the manuscript and did the statistical analysis.

**SHA:** Takes responsibility and is accountable for all aspects of the work in ensuring that questions related to the accuracy or integrity of any part of the work are appropriately investigated and resolved.

**HB:** Did data collection.
